# Light Emitting Marker for Robust Vision-Based On-The-Spot Bacterial Growth Detection

**DOI:** 10.3390/s17061459

**Published:** 2017-06-21

**Authors:** Kyukwang Kim, Jieum Hyun, Jessie S. Jeon

**Affiliations:** 1 Urban Robotics Laboratory, Korea Advanced Institute of Science and Technology, 291 Daehak-ro, Daejeon 34141, Korea; kkim0214@kaist.ac.kr (K.K.); jimi.hyun@kaist.ac.kr (J.H.); 2 Department of Mechanical Engineering, Korea Advanced Institute of Science and Technology, 291 Daehak-ro, Daejeon 34141, Korea

**Keywords:** bacterial growth, light emitting marker, LED marker, fast Fourier transformation, robust growth detection

## Abstract

Simple methods using the striped pattern paper marker and FFT (fast Fourier transformation) have been proposed as alternatives to measuring the optical density for determining the level of bacterial growth. The marker-based method can be easily automated, but due to image-processing-base of the method, the presence of light or the color of the culture broth can disturb the detection process. This paper proposes a modified version of marker-FFT-based growth detection that uses a light emitting diode (LED) array as a marker. Since the marker itself can emit the light, the measurements can be performed even when there is no light source or the bacteria are cultured in a large volume of darkly colored broth. In addition, an LED marker can function as a region of interest (ROI) indicator in the image. We expect that the proposed LED-based marker system will allow more robust growth detection compared to conventional methods.

## 1. Introduction

The importance of bacterial culture has been emphasized by many case studies in various fields. The low cost and simple procedure of bacterial culture enables various applications and experiments in the field, especially on-site experiments or laboratories in developing countries [1]. The key barrier in utilizing bacterial culture experiments is growth detection, as it requires measuring of the optical density (OD) [2], which is difficult to automate. OD requires careful tuning and setup as the distance and angle between the photo emitter and receiver is important. Many commercial OD machines use mostly cuvettes or fixed-size vessels for measurement, and therefore, it is impossible or very cumbersome to control a small volume of liquid during the culture experiment. Some automated culture and OD measurement devices are commercially available; however, these machines are generally expensive, not portable, and too sensitive for field operations.

To replace existing OD measurement methods, Kim et al. [3] proposed a visual marker-based solution to conduct bacterial growth measurement. This method uses a stripe patterned marker placed in the culture vessel and takes the image of the marker. As the bacteria grow and blur the liquid culture media, the fast Fourier transformation (FFT) spectrum decreases as the high frequency region of the image caused by the marker pattern disappears [4]. The resolution of this method was lower than that of the OD measurement, but was still able to clearly distinguish between the fully-grown state (near the end of the log phase of the growth curve: about 0.8~1.5 of OD_600_ value) and no-growth state (before the growth curve enters the log phase: OD_600_ value < 0.3). Since many bacterial detection methods just require Presence/Absence (P/A) results [2,5,6], this method can replace OD used in P/A tests and help automate the biological experiment procedures.

The key feature of the FFT based measurement is this method only requires appropriate image of the marker between the culture vessel regardless of the factors such as size of the reactor or distance of the marker and image sensor. Exact tuning of angle and distance of the photodiodes and the light emission power based on the turbidity and thickness of the vessel and contained liquid is not required in this case. Easy automation allows processing multiple remote samples at the given time; treating large number of samples at local public health authority in case of outbreak of contagious diseases or handling water samples at a large food processing facility [2]. Visual inspection by the technician can replace the checkup procedure; however, it becomes very labor intensive and error prone as the human inspector can easily make mistakes due to fatigue.

However, the application of the marker and FFT spectrum calculation has a few limitations. Since it is an image processing based technique, proper lighting is required. The bacterial culture processes are generally performed in the culture room or inside the incubator for 4~24 h and it is unrealistic to expect proper lighting conditions in the incubator or during an overnight culture. The second problem is the error caused by the color of the liquid broth. The frequently used liquid broths such as nutrient broth (NB) or Luria-Bertani broth (LB) have clear, weakly yellow colors. However some broths like tryptic soy broth (TSB) have dark brown colors. When culturing bacteria in a large volume of this dark medium, even before the bacteria has had time to grow, the dark color of the broth blocks the marker and hides the striped patterns, thus disrupting proper FFT calculation of the image.

**Figure 1:**
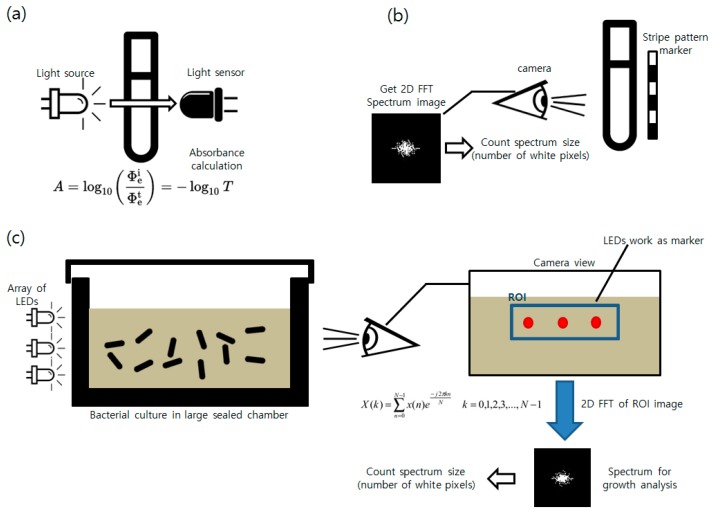
Illustration explaining basic principles of the bacterial growth measurement methods (**a**) optical density calculation using light absorbance, (**b**) FFT spectrum measurement using an image of the striped pattern marker over the culture vessel, (**c**) the proposed method using an LED array as a marker.

In this research, the authors propose a light emitting marker-based method to overcome the addressed problems of the existing marker system. We aim to build a system that can distinguish the growth state of the bacteria in the 7 cm thick rectangular vessel (distinguishing the OD_600_ value under 0.2 and over 0.7) regardless of the presence of the external lightings when culturing with the LB and TSB broth. Furthermore, we aim the final prototyping cost of the entire incubating system and image processing units to be less than 150 U.S. dollars to facilitate spread of similar systems to the users. Unlike in the striped pattern marker-based system, in the proposed system, the LED placed along the culture vessel emits the light used for the measurement. Instead of measuring the strength of the transmitted light as is done in the OD method, an image of the glowing LED itself is taken. Multiple LEDs are placed in series to create an array that has a similar configuration as the striped pattern marker. The repeating LEDs serve as both the region of interest (ROI) indicator and FFT pattern generator. Growth detection in an environment without LEDs or bacteria cultured in dark-colored broth was performed to show that, by comparison, the proposed LED array markers have higher robustness than the striped pattern marker. Moreover, a new incubator system with a simpler design and improved portability compared to existing systems [2] was developed. A schematic describing the process of the ODs, striped pattern marker, and the proposed LED array marker is shown in Figure 1.

## 2. Materials and Methods

### 2.1. System Overview

**Figure 2:**
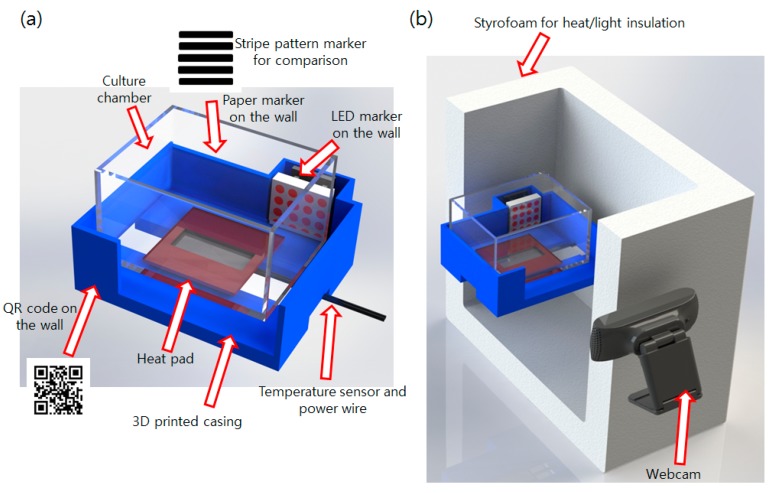
Developed incubator system (**a**) culture chamber with 3D-printed casing holding markers, sensors, and heat source; (**b**) final assembly of the culture device in the box with camera.

An electrically controlled incubator was developed to conduct the bacterial growth and detection. A 70 × 90 × 50 (mm) plastic airtight container which can hold about 50 mL volume of culture broth was used as the main culture vessel. The 3D-printed casing was designed and printed for attaching the sensors, heat source, and markers to the vessel. Since the bacterial culture chamber requires frequent washing and replacing owing to hygiene and biological safety requirements, instead of directly attaching devices to the culture vessel, all devices were attached to the casing and the culture vessel was placed in the casing. A 12 V 10 W low-powered silicon rubber heater was used as the heat source. Conventional systems [2] use household hot wire, which requires AC 220 V as the heat source and thus corded power for operation. This is not suitable for field operations. Thus, the developed incubator uses a battery-powered heat source to resolve this power issue. An Arduino Uno and LM35DZ sensor (Texas Instruments, Dallas, TX, USA) were used for the temperature control. The QR codes and the striped pattern markers were attached to the 3D-printed casing for comparison with the existing method [3] and the proposed method. A 4 × 4 LED matrix with 5 mm diameter LEDs was used as the LED array marker. The number of LEDs did not matter significantly. We used four LEDs to mimic the repeating stripe-like patterns used in the previous method. Too few LEDs would likely cause problems such as weak pixel differences (low frequency at default state). The assembled incubator was placed inside of a styrofoam box for heat insulation and background blocking. A webcam was placed about 23 cm far from the LEDs (distance does not matter if the acquired image contains all LEDs at the ROI) to monitor the incubator and search marker through a small hole in the box. The webcam is connected to an image processing unit such as a laptop or embedded Linux board such as the Raspberry Pi. A smartphone-laptop based image sending and analyzing system used at [3] is also available. The developed hardware system is shown in Figure 2.

### 2.2. Bacterial Strain Preparation

For the experiment, the *Escherichia coli* (*E. coli*) BL21 strain which was provided by the Analytical Biochemistry Laboratory of KAIST were used. The bacterial strain was thawed from the deep freezed stock and subcultured at the LB broth in the 37 °C incubator. The cells were cultured overnight (about 20 h) at the 5 mL LB broth in the shaking incubator before the final innoculation. The final OD_600_ value of the subcultured cell was 1.9. A 2 mL measure of the subcultured broth was innoculated to the 50 mL culture broth (TSB) in the main culture vessels (70 × 90 × 50 mm) and placed at the 37 °C incubator. The starting OD_600_ value after the innoculation to the 50 mL culture broth was 0.15.

### 2.3. Image Processing Algorithm

**Figure 3:**
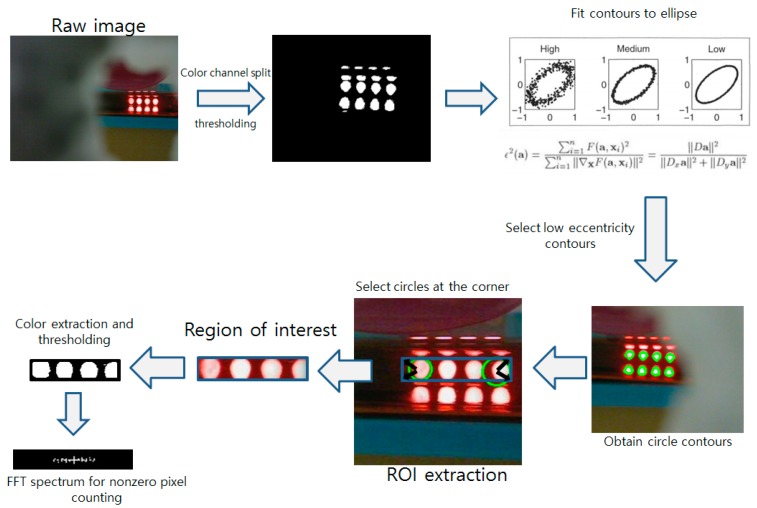
Flow chart describing the image processing pipeline developed in this research.

Image acquisition and processing were performed to detect the location of the LED array and check the turbidity of the broth. After a photo was taken by the attached webcam, the red channel was split since the LEDs had a bright red color. Appropriate thresholding, erosion, and dilation were performed to produce large contours only. All detected contours were processed by the Fizgibbon95 algorithm [7] (fitEllipse function of the OpenCV library [8]) and fitted into an ellipse. The eccentricity of each fitted ellipse was calculated and the contours with the low eccentricity, which means that they were more similar to a circle than of an ellipse, were selected. The Hough Circle Transform [9] is generally used for circle detection; however, this function is optimized for identifying perfect clear circles. Even though the LEDs of the array had circular shapes, the reflections and distortions caused by the broth and culture vessel slightly deformed the shape of the LED lights, making it difficult to detect using the Circle Transform. Usage of the eccentricity was a much more robust approach than the Hough Circle Transform, since the currently implemented version of the Hough Circle Transform requires at least five parameters and is more difficult to optimize compared to the eccentricity calculation, which only requires identifying the threshold of a single parameter. Among the detected circles, the circles with the largest and smallest x coordinate were selected (rightmost and leftmost). The four selected circles were filtered by the y coordinate value to pick the two both end circles at the upper row. Two lines with the angle of 45° and 135° for the right end center and 225° and 315° for the left end center were drawn from the center of the center. The cross section (the black arrows of the Figure 3 ROI extraction show the four corner selection) of the drawn line and the edge of the circles were selected as the four corners of the final ROI. The coordinates of the initial ROI were saved and further used. The spectrum size (number of nonzero pixels in the FFT spectrum image) of the ROI image section was calculated and used for the growth detection. The overall image processing procedures are described in Figure 3.

## 3. Results and Discussion

**Figure 4:**
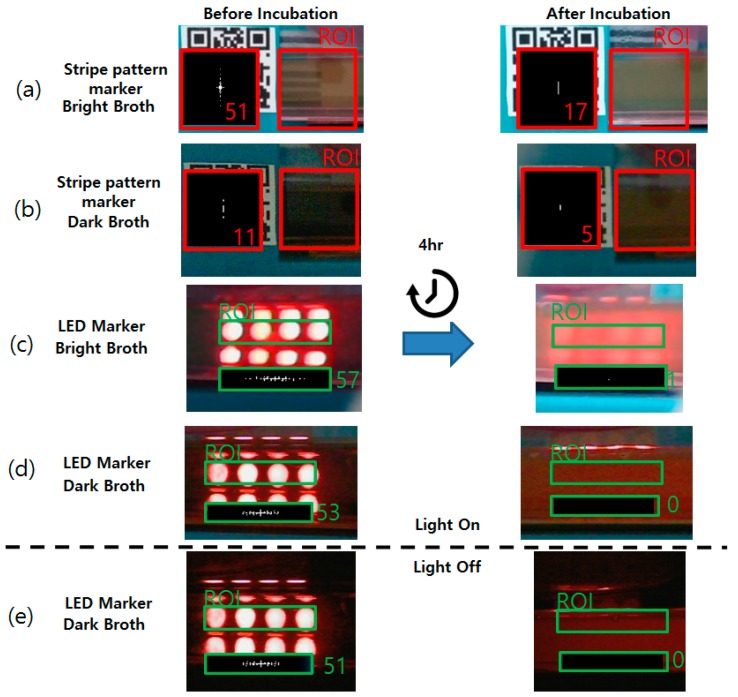
Growth measurement results comparison of the proposed LED array marker and printed striped pattern marker of the previous research in various visibility conditions. Red/green square box indicates the ROI (marked with the letter ROI) and the FFT pattern calculated from the ROI (marked with the number). The written number next to the spectrum indicates nonzero pixel size (FFT count). Results of before incubation (0 h) and after the incubation (4 h) were compared at conditions with (**a**) stripe pattern marker with the LB broth, (**b**) stripe pattern marker with the TSB broth, (**c**) LED marker with the LB broth, (**d**) LED marker with the TSB broth, and (**e**) LED marker with the TSB broth without the lightings.

Growth measurement tests were performed when the color of the culture broth was dark to evaluate the functionality of the proposed method. For comparison, the striped pattern marker method was also introduced under the same conditions. Images of each marker were taken at the point of inoculation and after 4 h of incubation. LB broth was used as the brightly colored broth and TSB broth was used as the dark-colored medium. The experimental results are shown in Figure 4.

As shown in Figure 4, the striped pattern marker was highly distinguishable in the brightly colored medium (Figure 4a). Previous research [3] reported a decrease in the FFT spectrum area after the bacteria had grown, and same results were replicated in this study. The FFT spectrum size (number of nonzero pixels) of the clear LB showed a count of 51, and dropped to 17 after being cultured. However, this difference in the FFT spectrum size did not show when the dark colored medium was used (Figure 4b). Even though the bacteria had not yet grown, the FFT spectrum size showed a value of only 11, much smaller than the value measured from the brightly colored broth. The culture vessel used in the previous research study used approximately 3 to 5 mL of broth in either a thin culture tube (radius of 20 mm) or in a millifluidic scale PDMS device [3]. Though dark broth was used, it did not significantly affect the measurements since the thickness of the liquid layer was too thin. However, environmental bacterial detection methods such as coliform detection [2] require a larger volume to process, which can be problematic in data acquisition as shown in Figure 4.

Compared to the striped pattern marker, the proposed LED marker showed similar visibility when the bright broth was used (Figure 4c), but showed better visibility when the dark broth was used (Figure 4d). Though the thickness of the dark liquid layer increased, the bright red circles were clearly observable. After 4 h of incubation, the blurred media concealed the glowing LEDs and only scattered red lights were seen. The FFT calculation showed 51 counts before the incubation and 0 counts after the culture was completed.

**Figure 5:**
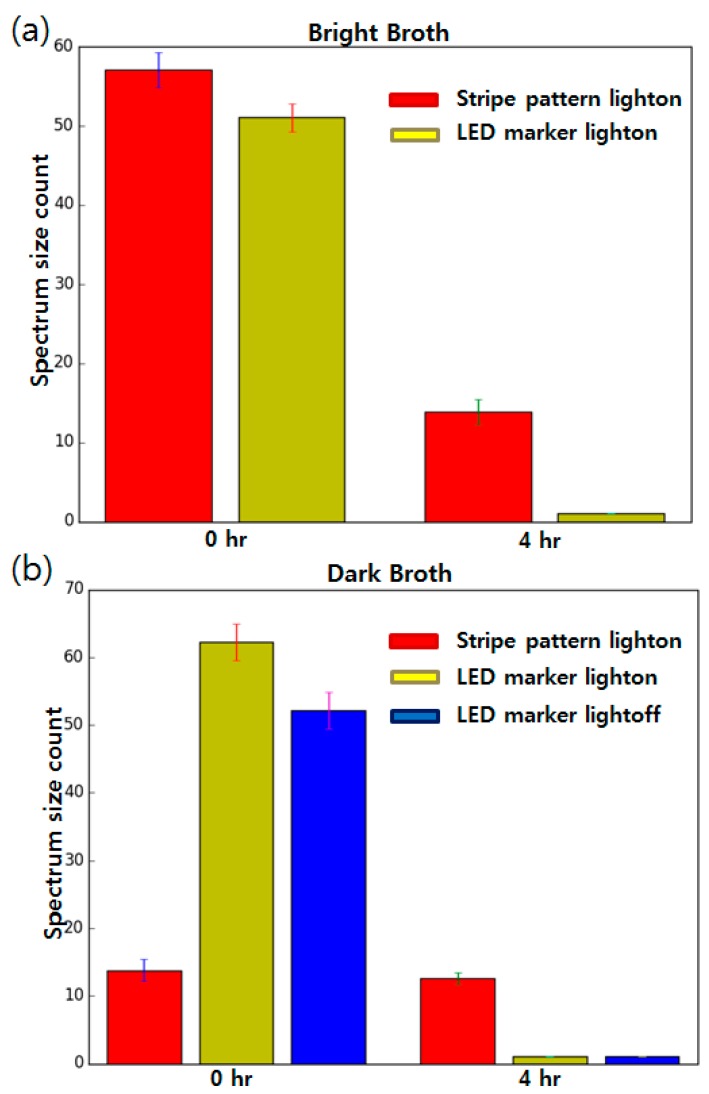
Plot showing the mean and standard error values of experiments done using bright broth (**a**) and dark broth (**b**). (*n* = 5) Data points having a value of 0 were converted to 0.5 during plotting for better visibility. The y axis indicates the number of nonzero pixels in the FFT spectrum images.

Moreover, the consistency of the LED array marker FFT calculation results was maintained, even when the lighting was changed. The culture box was placed in a completely dark room and the incubation was performed. The results of the zeroth hour and fourth hour (Figure 4e) showed no significant difference from the results measured with light (Figure 4d). The FFT spectrum at the zeroth hour yielded counts of 51 (53 in the bright room) and 0 (0 in the bright room) after the fourth hour. Obviously, the striped pattern paper marker was not observable when no light was provided. Multiple FFT spectrum size values were collected from each condition and compared. The results are summarized in Figure 5.

In the brightly colored broth, both the striped pattern marker and LED marker showed similar results; high FFT counts at the zeroth hour and low FFT counts at the fourth hour were observed. However, previous research studies could not distinguish the zeroth hour from the fourth hour when using dark broth. The average FFT count value showed only a 0.8 point difference while more than 20 count differences could be observed when the brightly colored broth was used. However, the proposed LED marker still showed good detection ability, even when the dark colored broth was used with about 60 FFT count differences. In the dark environment without lighting, the striped marker imaging was not possible, whereas the LED marker still functioned with similar FFT count differences to experiments with other conditions. The results show that it is expected that the proposed method can be effectively used in P/A tests such as antibiotics susceptibility tests (AST) or toxicity measurements [10,11,12], where comparison of only the growth/no growth state is required.

**Figure 6:**
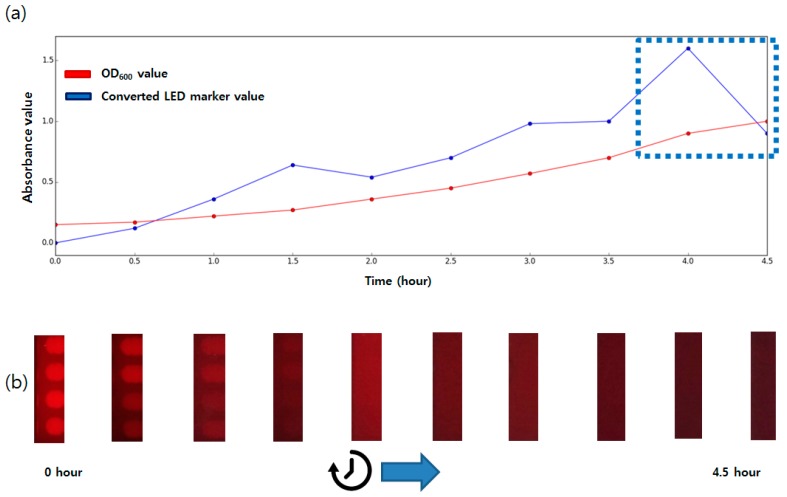
(**a**) OD600 value and absorbance FFT spectrum size during the measurement time. (**b**) LED ROI images at each measurement time.

For the comparison with the traditional technique, OD_600_ (OD at the 600 nm wavelength) values were also measured with the commercial spectrometer. (Ultrospec 7000, GE, Boston, MA, USA). From the inoculation, OD values and sensor outputs from the proposed method were measured every 30 min until the OD value reached 1.0. Light was turned on during the measurement as the proposed LED marker system does not affect by the presence of the lighting as shown at the Figure 5. For easier comparison, the FFT values were log transformed using the following absorbance calculation formula,

(1) $$A=-\log_{10}(T_{i}/T_{t})$$

where measured spectrum area count of the sample was used as *T_i_* value and the maximum area value was used as *T_t_* value [3]. The data are shown at the Figure 6.

The both measurement values increased as time passed; the OD value changed from 0.15 to 1.0 and the FFT spectrum size changed from 0 to 1.0. Two data showed good correlation (*r*^2^ value of 0.97 with *p*-value < 0.01 from the Pearson correlation analysis) which indicates that the LED marker based measurement can be used for the bacterial growth measurement in the liquid medium. Compared to the optical density, the proposed method showed rather rapid increase at the early stage of the incubation (0 to 1.5 h). Towards the end of the culture period, the output of the sensor became noisy due to saturation at the near OD 0.9 (dotted box at the Figure 6). At the high cell density, the FFT spectrum size drops to near 0, which gradually increases the absorbance which is the negative of log transformed value. The cuvette used for the OD measurement contains only 1 mL of the broth between thin container walls while the main culture vessel of the proposed system is 70 mm thick. Although there is small increase of the bacterial cells in each unit of volume of the culture broth, the containers with thick walls are more affected by these changes as the total number of the integrated bacterial cells between the LED marker and camera are larger than the small containers such as cuvette. We expect that the changing the size of the culture vessel can help in adjusting the measurement resolutions or measurement range of the proposed system. For the target application of this article, the P/A test, where early detection of the meaningful bacterial cell increase is important, a thick container which allows faster turbidity increase of the culture vessel could be an appropriate selection. If a user wishes to obtain higher resolution data at high concentrations (e.g., culture until OD 1.0 for competent cell preparation), it would be desirable to use a small container such as a 50 mL tube.

The overall results showed that the LED array marker functions under conditions in which obtaining a clear image of the striped pattern marker is difficult or impossible. Consistent lighting is very important for the image processing, but it is often challenging to establish the proper settings. On-the-spot tasks, requiring the handling of environmental samples in an outdoor environment, must conceal sunlight while installing the appropriate light settings in the incubator for proper illumination. Laboratories can rather easily set the light conditions, but operating the lights during the entire culture time (4 h to overnight) is a waste of electricity, especially in places where the electrical infrastructure is not well established. Even when light sources are installed in the incubator box, reflections of the light to the nearby surfaces can disturb proper image processing. The proposed method uses a light emitting marker to solve this issue. Since red LEDs generate higher intensity compared to other objects, they are easily detectable in the noisy environment or behind the larger culture chamber. Even when the external lights are turned off, they can still function since the marker itself is a light source. Additionally, the camera used in this research is a low cost webcam, which is much cheaper and easier to connect to various devices such as a laptop compared to the smartphone camera used in the previous research. Bright and easy detectability of the LED marker can help decrease the total cost of the system and improve the interfacing between devices.

The incubator system used to obtain automatic measurements was also improved compared to previous research investigations. Previous studies used a small culture tube attached to a shaking incubator, which required a 220 V hot wire to warm up the entire incubator box. The newly developed incubator uses a low powered heating pad directly attached to the culture chamber; it decreases the amount of energy required to warm up the culture chamber. Also, removing the actuating component helps to decrease the weight and power consumption. The proposed system improves image processing, lowers energy consumption, and enables the use of a larger culture volume, allowing for portable bacterial culture. These features can facilitate starting the culture right after the sample is collected or during its transport to the laboratories. Pathogens such as *Vibrio cholera*, *Vibrio vulnificus*, or *Vibrio pharahaemolyticus* [13,14,15], which are found in warm seawater, require enrichment processes before the separation or identification is performed [1]. Multiple culture steps take a long time and make detecting these pathogens labor intensive. Being able to start the culture and growth check of these pathogens during transportation can help decrease the total time required to conduct the bacterial experiments.

## 4. Conclusions

This paper proposes an advanced version of the vision marker-based bacterial growth measurement method. An LED array was used instead of a paper marker, which cannot be detected when the lighting is too dim or the color of the broth is dark. In addition, the previous method required a QR code to obtain the ROI within the image, but the LED array of the proposed method works as both a marker and a position indicator. The experimental results showed that the LED markers can function in harsh environments compared to the paper markers. An upgraded version of the existing bacterial incubator which costs only about 130 U.S. dollars was also introduced to resolve power issues associated with it. The cost includes incubating units and computing systems and multiple incubating units can be connected to the single computing units to lower the costs for preparing multiple units.

In future works, we will consider encoding information into the LED markers. The QR code of the previous research functions as the ROI indicator, but information such as culture vessel identification (ID) can also be encoded. If data codes such as hamming markers or AprilTags can be imported into the LED marker, then the marker can additionally serve as an information source as well as an indicator and position identifier.
